# Establishing the Role of Elbow Muscles by Evaluating Muscle Activation and Co-contraction Levels at Maximal External Rotation in Fastball Pitching

**DOI:** 10.3389/fspor.2021.698592

**Published:** 2021-11-30

**Authors:** Bart van Trigt, Eva Galjee, Marco J. M. Hoozemans, Frans C. T. van der Helm, DirkJan H. E. J. Veeger

**Affiliations:** ^1^Department of Biomechanical Engineering, Delft University of Technology, Delft, Netherlands; ^2^Department of Human Movement Sciences, Faculty of Behavioural and Movement Sciences, Vrije Universiteit Amsterdam, Amsterdam Movement Sciences, Amsterdam, Netherlands

**Keywords:** electromyography, musculoskeletal injuries, Tommy John surgery, overhead sports, ulnar collateral ligament (UCL), injury prevention, baseball

## Abstract

**Background:** Baseball pitching is associated with a high prevalence of ulnar collateral ligament injuries, potentially due to the high external valgus load on the medial side of the elbow at the instant of maximal shoulder external rotation (MER). *In-vitro* studies show that external valgus torque is resisted by the ulnar collateral ligament but could also be compensated by elbow muscles. As the potential active contribution of these muscles in counteracting external valgus load during baseball pitching is unknown, the aim of this study is to determine whether and to what extent the elbow muscles are active at and around MER during a fastball pitch in baseball.

**Methods:** Eleven uninjured pitchers threw 15 fastball pitches. Surface electromyography of six muscles crossing the elbow were measured at 2000 Hz. Electromyography signals were normalized to maximal activity values. Co-contraction index (CCI) was calculated between two pairs of the flexor and extensor elbow muscles. Confidence intervals were calculated at the instant of MER. Four ranges of muscle activity were considered; 0–20% was considered low; 21–40% moderate; 41–60% high and over 60% as very high. To determine MER, the pitching motion was captured with a highspeed camera at 240 Hz.

**Results:** The flexor pronator mass, pronator teres, triceps brachii, biceps brachii, extensor supinator mass and anconeus show moderate activity at MER. Considerable variation between participants was found in all muscles. The CCI revealed co-contraction of the two flexor-extensor muscle pairs at MER.

**Interpretation:** The muscle activation of the flexor and pronator muscles at MER indicates a direct contribution of forearm muscles crossing the medial side of the elbow in counteracting the external valgus load during fastball pitching. The activation of both flexor and extensor muscles indicates an in-direct contributory effect as the combined activity of these muscles counteract opening of the humeroulnar joint space. We believe that active muscular contributions counteracting the elbow valgus torque can be presumed to relieve the ulnar collateral ligament from maximal stress and are thus of importance in injury risk assessment in fastball pitching in baseball.

## Introduction

Baseball pitching is a sports action that stresses the medial side of the elbow and is associated with a high prevalence of medial elbow injuries (Olsen et al., 2006; Conte et al., 2015). The current leader of medial elbow injuries in pitchers is an injury to the medial Ulnar Collateral Ligament (UCL) with 25% of the Major League Baseball pitchers having undergone UCL reconstruction during their career (Conte et al., 2015). It is desired to prevent pitchers from experiencing UCL injuries to save associated costs and increase playability. Understanding the pathophysiological mechanisms through mechanical analyses of sustaining an elbow injury, and more specifically an injury to the UCL, might shed light on effective injury prevention programs.

Inverse dynamics studies show that, when performing a baseball pitch, shortly before shoulder maximal external rotation (MER), as the throwing arm transitions through the arm cocking phase and acceleration phase, the elbow resists its peak load (Werner et al., 1993; Fleisig et al., 1995; Gasparutto et al., 2016). At this instant the elbow is exposed to an external valgus torque of reportedly 60–120 Nm. It is stated that the external valgus torque at the timing of MER is identified as a critical load related to medial elbow injuries (Fleisig et al., 1995). The external valgus torque can be resisted by structural stabilizers, such as joint articulations and ligaments. According to *in-vitro* studies the anterior band of the UCL is the main structural stabilizer capable of resisting an external valgus torque (Kaufmann et al., 2019). In addition, it has been reported that pitchers throwing with a higher external valgus torque have a thicker UCL compared to pitchers who throw with a lower external valgus torque (Hurd et al., 2011), indicating that the UCL is important in resisting the external valgus torque. However, the precise relationship between external valgus torque, UCL load, UCL characteristics and UCL injuries in baseball pitching is unknown.

The literature shows that not only the UCL but also functional stabilizers, such as muscles, are able to counteract the external valgus torques, either direct or indirect (Van Trigt et al., 2021). *In vitro* studies show that the flexor pronator muscle group (FPM), which consists of the m. pronator teres, m. digitorum superficialis, m. flexor carpi ulnaris and the m. flexor carpi radialis, is a significant contributor to counteract an external valgus torque (Park and Ahmad, 2004; Lin et al., 2007; Seiber et al., 2009; Udall et al., 2009). The forearm flexor muscles could have a direct effect in counteracting the external valgus torque during pitching. In addition, the interaction between the functional stabilizers and the elbow joint geometry could have an indirect effect on the valgus torque by increasing the joint compression force (Van Trigt et al., 2021). Several *in-vitro* studies showed that simulated loading of the biceps and triceps brachii significantly decreased the ulnohumeral joint space and thus resist the external valgus torque (Morrey et al., 1991; Seiber et al., 2009). In addition, a forward dynamic musculoskeletal model showed that simulated activation of the triceps brachii and biceps brachii increased joint contact force (Rahman et al., 2018). We therefore assume that co-contraction of flexor and extensor elbow muscles could indirectly counteract the external valgus torque indicating an indirect effect. Hence, the biceps, triceps, anconeus and ESM cannot provide direct stability, like the FPM. However, it is unknown whether elbow muscles are active at the instant of MER during pitching and thus can, either directly or indirectly, counteract the external valgus torque.

Electromyography (EMG) studies measured the activity of the elbow muscles during baseball pitching, in either the cocking or acceleration phase of the pitch. Activation of the FPM, biceps and triceps was found in all studies (Sisto et al., 1987; Digiovine et al., 1992; Glousman et al., 1992). These studies suggest that the muscles in the throwing arm are active at the late cocking and acceleration phase, which includes the critical instant of MER. Unfortunately, all studies averaged the EMG activity over each pitch phase, resulting in limited information on the activation pattern of the muscles potentially related to counteracting the external valgus torque at MER. More detailed EMG data are essential to investigate whether muscles contribute to counteracting the external valgus torque during a fastball pitch.

Therefore, the aim of this study is to determine whether and which elbow muscles show activity at MER during a fastball pitch in baseball pitchers. It is hypothesized that: (1) For a direct effect, elbow muscle activation is expected at the instant of MER for the FPM and PT, (2) For an indirect effect, co-contraction of flexor and extensor elbow muscles is expected at the instant of MER.

## Methods

### Participants

Eleven experienced male pitchers, with a mean age of 27 (SD 10) years, a mean body height of 1.87 (SD 0.08) m and a mean body mass of 87.4 (SD 17.9) kg participated in this study. Eight pitchers threw right-handed and three left-handed. They started playing baseball at a mean age of 7 (SD 2) years and started pitching at a mean age of 11 (SD 5) years. During the experiments they threw at a mean ball speed of 67 mph (29.95 m/s) [SD 7 mph (3.13 m/s)]. Two pitchers are playing at the highest level in the Netherlands, three pitchers at the second highest level and the other pitchers at amateur level. At and in the 6 months prior to the measurements all participants reported to not have experienced musculoskeletal injuries. This research was conducted in accordance with the Declaration of Helsinki and the local ethics committee of the Technical University Delft approved the research protocol. Informed consent was obtained from all participants after being informed of the procedure of the study.

### Procedure

The measurements were performed at indoor facilities. Prior to performing fastball pitches, participants had to perform maximum voluntary contractions (MVC) in accordance with the functional characteristics of the muscles (see Supplementary Table A.1), of which the activity was recorded using surface electromyography (EMG). Participants had to slowly increase the force to a maximum effort within 3 s, hold it for 3 s and relax in 3 s again. Each specific MVC was repeated three times, with 30 s rest in between. After this, the participants were given an unlimited amount of time to physically warm-up before pitching fastballs at maximum effort. A pitching mound was installed from which the participants had to throw their pitches to a marked strike zone in a net, which was set at the regular pitching distance of 18.3 m from the pitching rubber. The participants were instructed to wear their own preferred clothes and baseball glove, but without a shirt during the measurements to avoid interference of the EMG signals. They had to perform 15 consecutive fastball pitches at maximum effort for data collection. The participant decided when ready to perform the next pitch, the rest was at least 30 s.

### Data Acquisition

Bipolar surface EMG of six skeletal muscles of the throwing arm was recorded from the flexor-pronator mass (FPM), extensor-supinator mass (ESM), pronator teres (PT), anconeus (Anc), biceps brachii (Bic) and lateral head of the triceps brachii (Tri) (Table 1). Because it is difficult to measure the activity of the wrist flexor muscles individually using surface EMG, we measured the activity of the forearm muscles combined as the FPM and the ESM. A reference electrode was placed at the spinous process of the 7th cervical vertebrae. Electrodes were placed based on the SENIAM guidelines (Hermens et al., 1999). After skin preparation, bipolar, disposable, pre-gelled Ag/AgCl surface electrodes (Blue Sensor Electrodes N-00-S, Ambu Inc., USA) were placed on the pitchers' skin with a gel-skin contact area of 1 cm^2^ for each electrode and an inter-electrode distance of 20 mm. The skin was shaved and cleaned with alcohol before the electrode attachment and the electrode cables were fixated to the skin to avoid cable movement artifacts in the signal and to minimize the risk of loosening of the electrodes from the skin during the pitch movement. The cables of the electrodes were connected to the bipolar active sensor BioPlux research device (Plux biosignals, Arruda dos Vinhos, Portugal), with 16 bits analog channels, a gain of 506 and an analog 25–500 Hz band-pass filter. Data were sampled at a frequency of 2000 Hz. All fifteen consecutive fastball pitches for each participant were recorded in one EMG dataset and locally stored on the BioPlux research device. A LED was attached to one of the channels of the BioPlux research device to annotate each throw and to synchronize EMG with kinematic data.

**Table 1:** Electrode position and orientation.

**Muscle (group)**	**Electrode position and orientation**	**Electrode placement**
m. biceps brachii (Bic)	On the line between the medial acromion and the fossa cubit at 1/3 proximal from the fossa cubit.	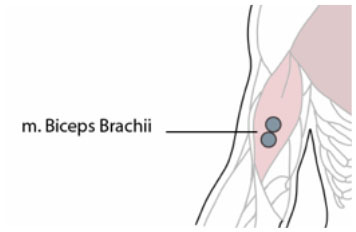
Flexor Pronator Mass (FPM)	At 1/3 distal from the medial epicondyle. In the direction of the line between the medial epicondyle and the middle of the radial and ulna styloi	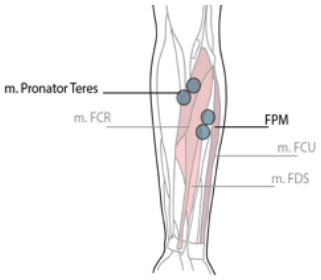
m. pronator teres (PT)	At 1/3 distal from the elbow joint between the medial and lateral epicondyle of the humerus. In the direction of the line between the medial side of the elbow and the lateral surface of the radius.	
m. triceps brachii (Tri) (lateral head)	At 1/2 on the line between the posterior crista of the acromion and the olecranon at two finger widths lateral to the line.	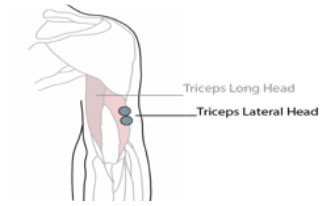
m. anconeus (Anc)	Parallel to and below the olecranon on the radial side. In line between the lateral epicondyle of the humerus and the ulna	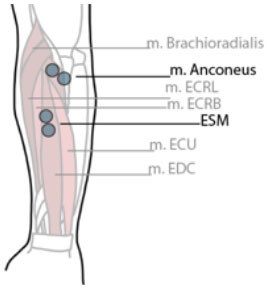
Extensor Supinator Mass (ESM)	At 1/3 distal from the lateral epicondyle of the humerus. In the direction of the line between the lateral supracondylar ridge of the humerus and the middle posterior side of the wrist.	

Kinematic data were collected with a high-speed video camera (Sony RX100V, Tokyo, Japan) at 240 Hz. The video camera was placed sideways relative to the pitching mound (camera height: 1.25 m, distance to mound: ±3.80 m). Ball speed of each pitch was recorded from behind the net at home plate distance with a Stalker pro radar gun (Stalker Radar, Plano, TX, USA).

### Data Analysis

#### Kinematics

To synchronize the kinematics with the EMG signals, videos were cut at the onset of the LED light. Video samples at the instant of foot contact (FC), maximal external rotation (MER) and ball release (BR) were visually determined for each pitch using Tracker (version 5.1.3, Open Source Physics). FC was defined as the moment that the foot of leading leg was in contact with the mound, MER was defined as the instant that the shoulder transitioned from an external to an internal rotation and BR was defined as the moment that the pitcher released the ball. The three pitch events were multiplied with an 8.33 (2000 Hz/240 Hz) sample rate conversion to correspond with the EMG signals.

#### EMG

EMG signals were cut into the 15 separate pitches using the block signal of 1.5 V of the LED flashlight. The EMG signal of each muscle within the 15 consecutive pitches was synchronized to the time of MER and cut at 600 samples (0.300 s) prior and 300 samples post MER (0.150 s), resulting in 15 pitch signal windows of 450 ms for six muscles per participant. The EMG pitch signals and MVC signals were concatenated for each muscle. An EMG linear envelope was obtained by rectifying the EMG using the absolute values of the Hilbert transform (Myers et al., 2003) and applying a fourth-order bi-directional low-pass Butterworth filter of 40 Hz. EMG data were normalized to the highest value of the concatenated filtered linear envelope signal (including both MVC and pitch data) for each muscle. Because EMG data of dynamic movements exceeds the MVC (Ball and Scurr, 2013), we decided to normalize the data to the highest obtained EMG value from either the MVC or pitch data. So, the EMG data does not exceed the 100%. In line with the study of Cavanagh and Komi (1979), normalized EMG data were time shifted relative to the kinematic data with 50 ms to compensate for the electromechanical delay (EMD). Thus, the results represented the muscle activity as an indication of the timing of relative muscle force. To quantify the in-direct effect, a co-contraction index (CCI) was calculated for two muscle pairs (biceps-triceps and FPM-ESM) according to Rudolph et al. (2000):


(1) $$CCI\;=\;\frac{EMG_{low}}{EMG_{high}}\;*\;(EMG_{low}\;+EMG_{high})$$

EMG_low_ is the normalized magnitude of the EMG signal for the less active muscle and EMG_high_ for the more active muscle. The CCI index can range from value zero (no co-contraction at all) to two (maximal co-contraction). All EMG data analyses were performed in Python (version 3.7, Python Software Foundation, https://www.python.org/).

### Statistical Analysis

From the 15 throws mean and standard deviation of the six normalized EMG signals were visualized over time. To visualize the in-direct effect, the EMG signals of the flexor muscles were labeled as positive and the extensor muscles as negative. In addition, the CCI were visualized over time. At the time instant of MER, the magnitude of the normalized EMG and CCI data was obtained. The assumption of normality was checked with the Shapiro-Wilk test of the EMG data at MER. On group level mean and 95% confidence intervals at MER were calculated to investigate if muscle activity were statistically different from zero. Within subject variability was defined as the 95 % confidence intervals calculated over the 15 throws. According to Digiovine et al. (1992), four ranges of muscle activity were considered to what extent muscles showed activity. A range of 0–20% was considered low; 21–40% moderate; 41–60% high and over 60% as very high.

## Results

After visually analyzing signals for artifacts, for instance due to loosening of electrodes, 74 (of the 990) signals from nine (of the 11) participants were excluded from the analysis. All EMG and CCI data at MER were normally distributed.

### Direct Effect

**Figure 1:**
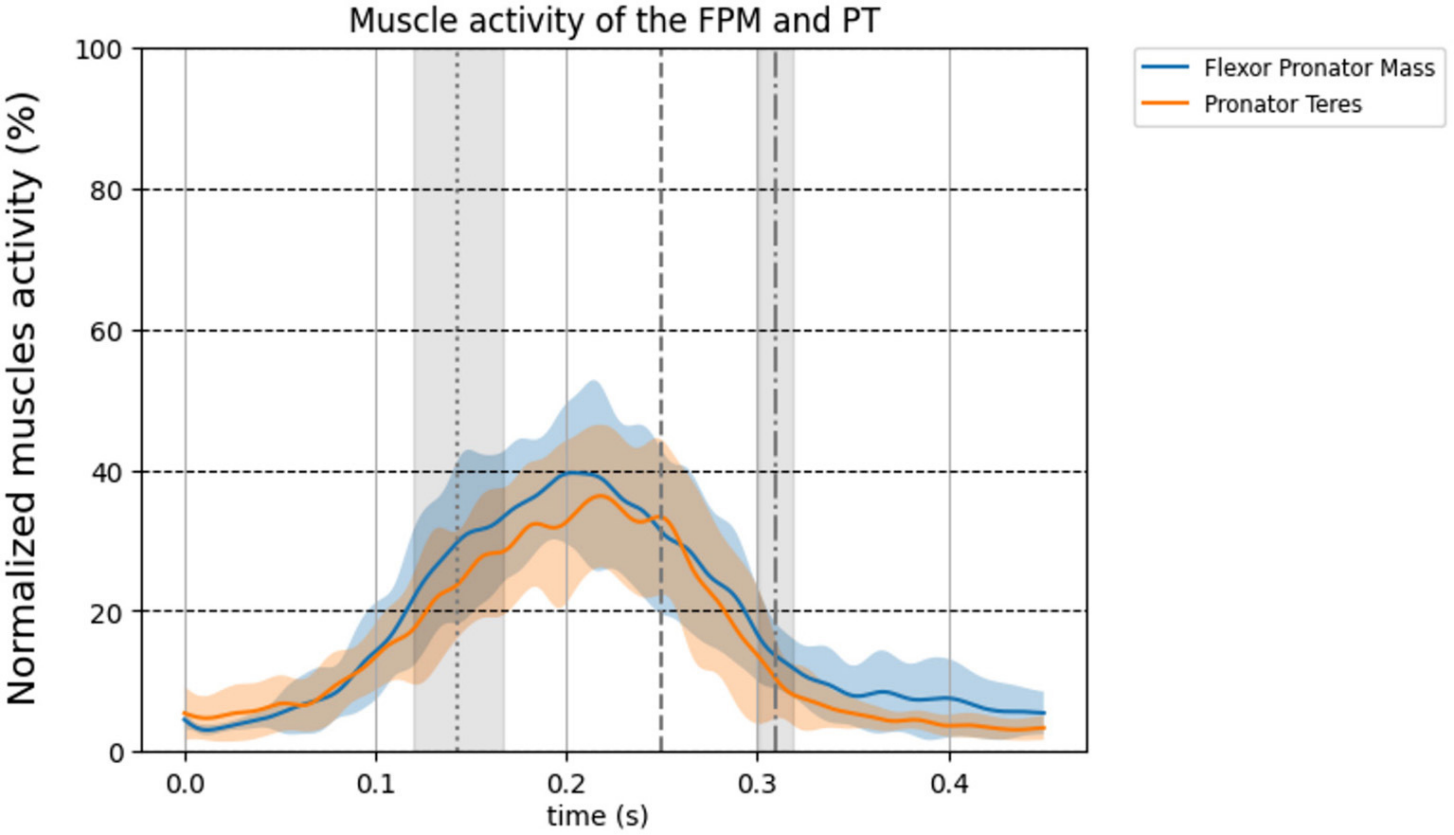
Normalized group level EMG signal time-series for the forearm muscles corrected for EMD (50 ms). The colored thick line in the time series shows the mean over all 11 pitchers and the standard deviation is shown as transparent area around the mean. Blue line: flexor pronator mass, orange line: pronator teres. The three vertical lines represent foot contact (dotted), MER (dashed), and ball release (dot dashed), respectively.

The normalized EMG data of the flexor lower arm muscles on group level are shown in Figure 1. Visual inspection shows activity of the FPM and PT at the instant of MER. On group level at the instant of MER the average FPM activity is 30.70%, 95% CI: [23, 39] and the average PT activity is 33.0%, 95% CI [25, 41].

### In-direct Effect

**Figure 2:**
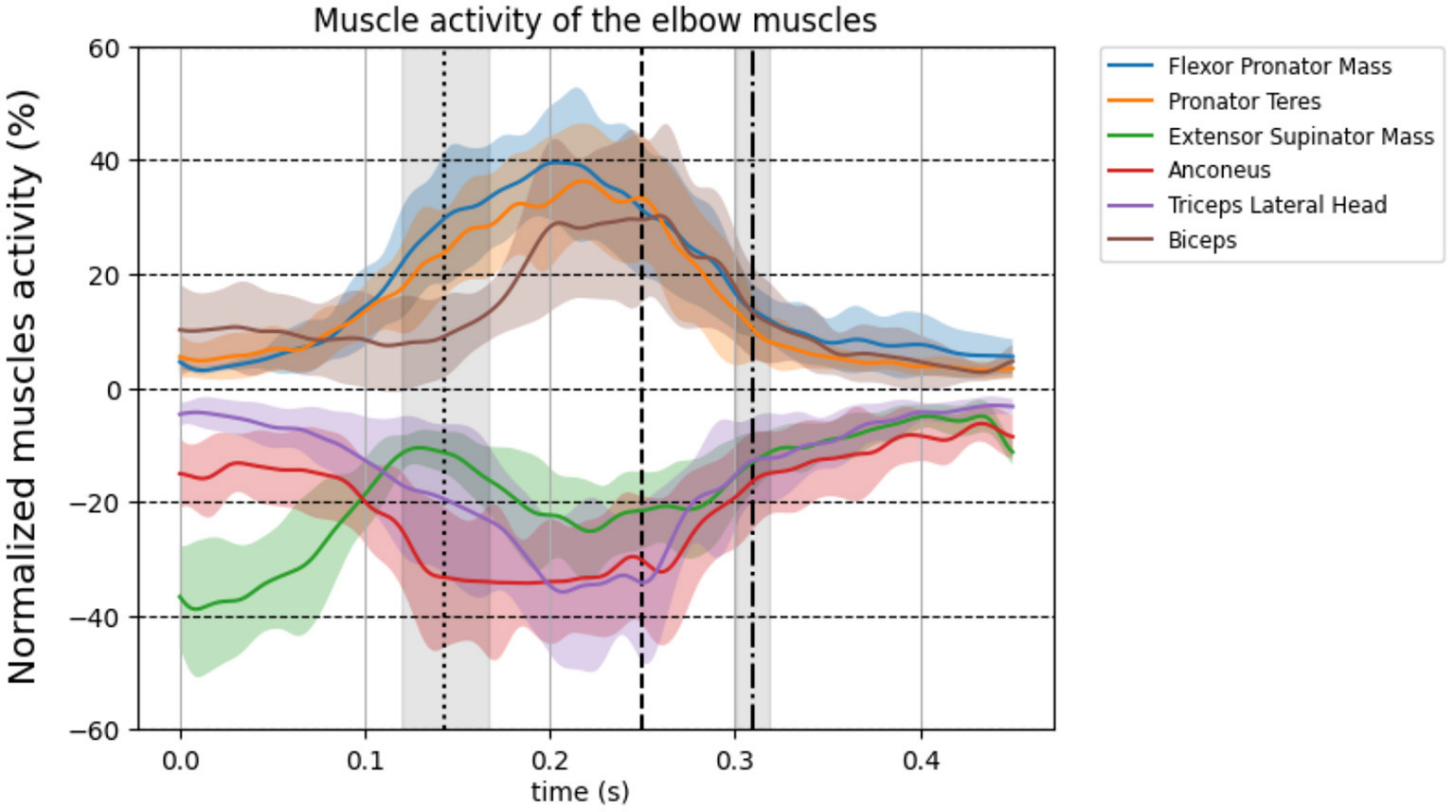
Group level normalized EMG signal time-series for the upper arm muscles corrected for EMD (50 ms). The thick line shows the mean over all 11 participants and standard deviation is shown as transparent area around the mean. The flexors are plotted positively on the vertical axis and the extensors are plotted negatively on the vertical axis. Brown line: biceps brachii, blue line: Flexor pronator mass, orange line: pronator teres, green line: extensor supinator mass, purple line: triceps brachii, red line: anconeus. The three vertical lines represent FC (dotted), MER (dashed), and BR (dot dashed), respectively. Be aware: the normalized activity ranges from 0% till 100%, here the y-axis scale ranges from 0% to 60% for better visualization.

**Figure 3:**
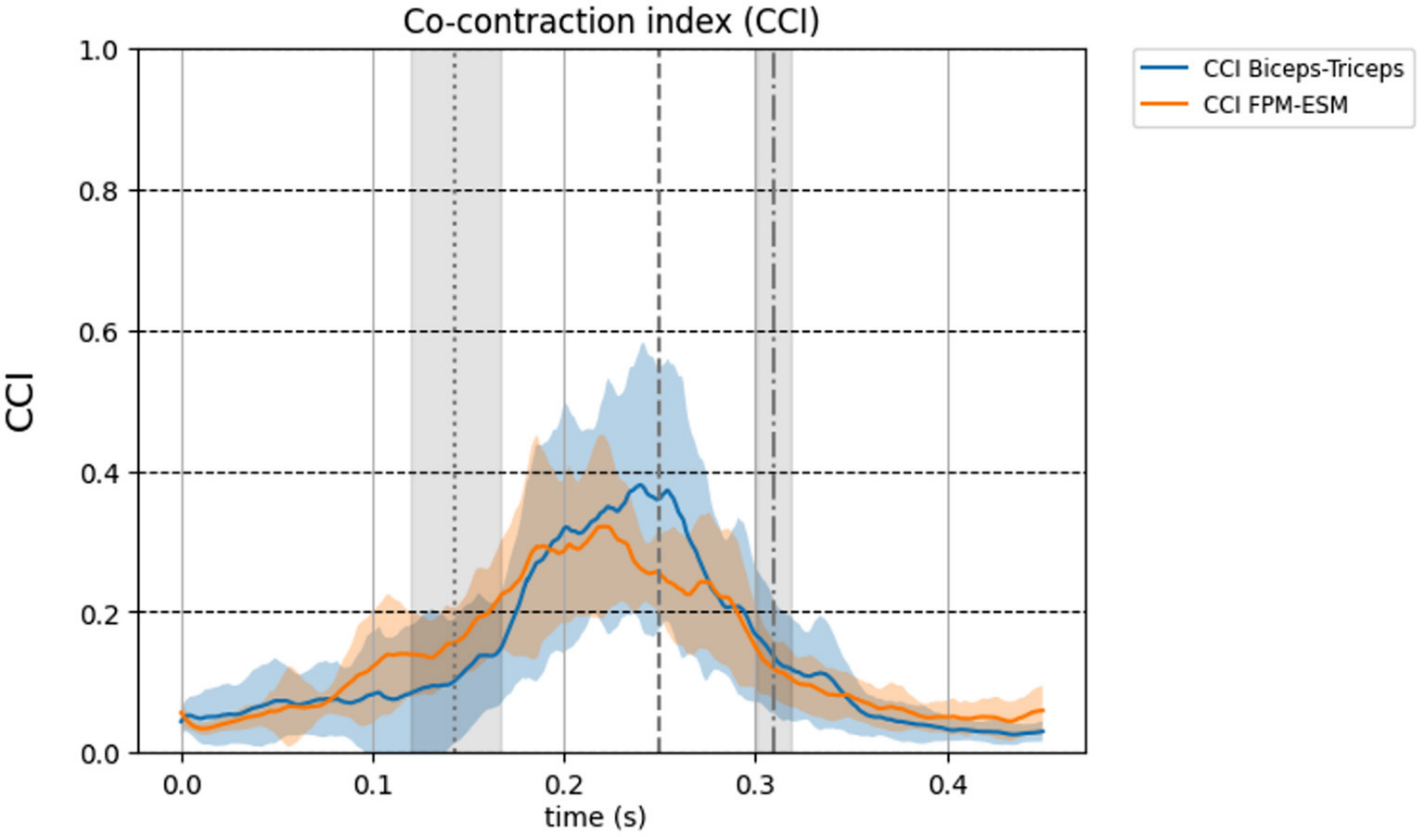
Group level of the co-contraction index of the elbow muscles during pitching corrected for EMD (50 ms). The thick line shows the mean over all 11 participants and standard deviation is shown as transparent area around the mean. Blue line: CCI Biceps-Triceps, orange line: CCI FPM-ESM. The three vertical lines represent FC (dotted), MER (dashed), and BR (dot dashed), respectively. Be aware: the co-contraction index ranges from 0 till 2, here the y-axis scale ranges from 0 to 1 for better visualization.

All EMG data of the flexor and extensor elbow muscles are shown in Figure 2. All muscles show maximal activity between FC and BR, except for the ESM which is most active before FC. Visual inspection shows elbow muscle activity of both flexor and extensor muscles simultaneously at the instant of MER on group level. On group level the average muscle activity at MER of the biceps was 29.8%, 95% CI [20.0, 39.7], the triceps was 33.5%, 95% CI [24.5, 42.5], the ESM was 24.4%, 95% CI [16.6, 26.2] and the anconeus was 33.7%, 95% CI [26.4, 41.0]. Figure 3 shows the CCI of the biceps-triceps and FPM-ESM during pitching. Visual inspection shows co-contraction at the instant of MER for both pairs. On group level the average CCI was 0.35, 95% CI [0.24, 0.47] for the biceps-triceps and 0.26, 95% CI [0.21, 0.30]) for the FPM-ESM at the instant of MER.

### Mean and Within Subject Variability

**Figure 4:**
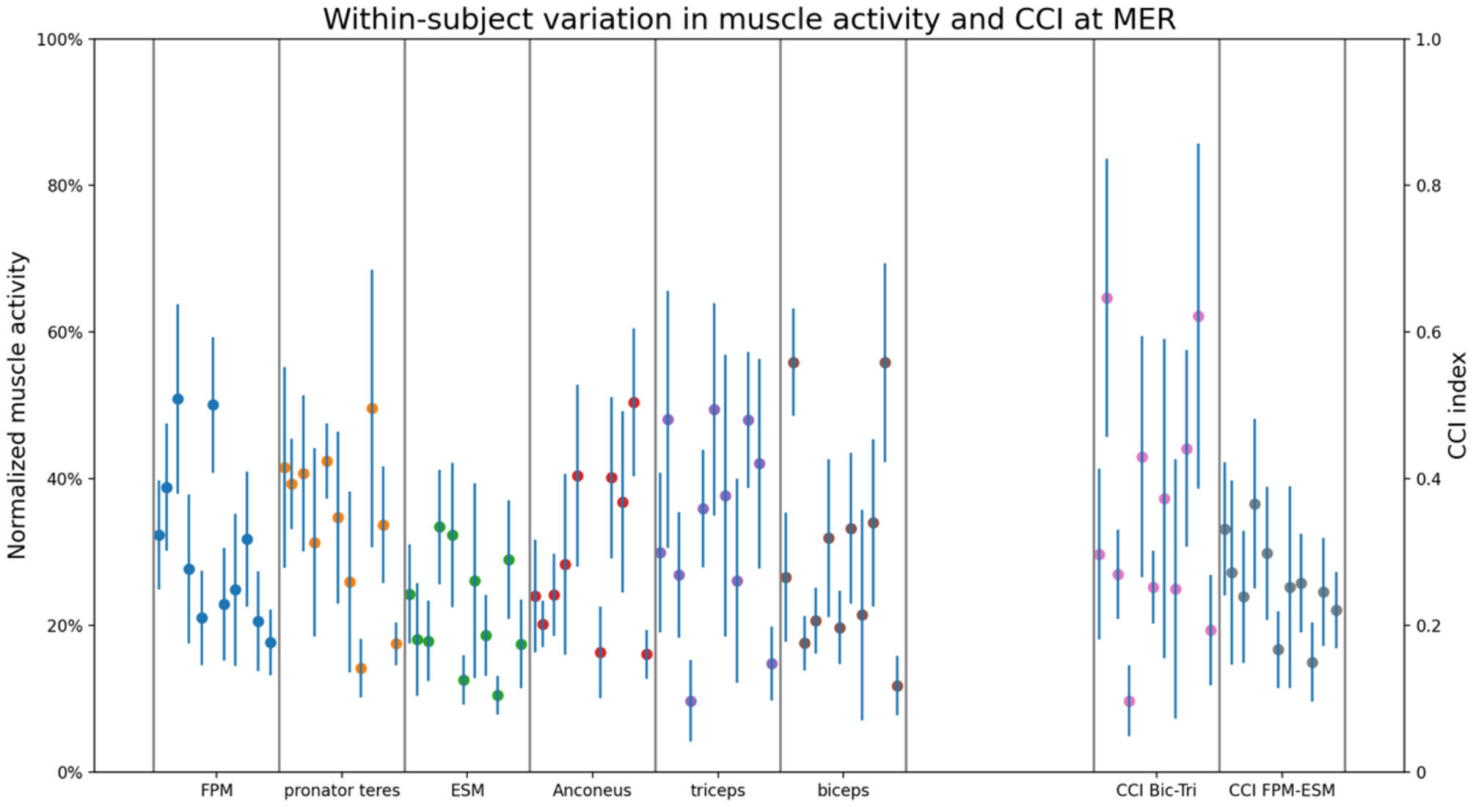
The within-subject variation in muscle activity and CCI for all measured muscles and CCI pairs at MER. Blue vertical lines show the confidence intervals calculated over the 15 thrown pitches. Each blue vertical line within one box (divided by the gray lines), represents the within-subject variation of each individual participant. The dots represent the mean muscle activity of the 15 thrown pitches for each participant.

Figure 4 shows the mean and confidence intervals of each participant for each muscle and CCI at MER. The dots between the two gray vertical lines represent the mean muscle activity for each pitcher. The blue vertical lines show the confidence intervals, representing the within subject variability.

## Discussion

The aim of this study was to determine whether and which elbow muscles show activity at MER during a fastball pitch, potentially to (partly) counteract the peak external valgus torque at the instant of MER. Moderate activity is observed in the FPM and the PT, indicating a direct effect. Elbow flexors and extensors are active simultaneously at the instant of MER, indicating an indirect effect. However, the flexor- and extensor muscle activity at MER is different between pitchers, resulting in wide ranges of muscle activity and co-contraction index values.

*In-vitro* studies show that the flexor pronator muscles are able to resist an external valgus torque at the elbow (Park and Ahmad, 2004; Seiber et al., 2009; Udall et al., 2009), although no reports are available describing whether these muscles are actually active at the relevant instant during the relevant pitch phase, i.e., at peak external valgus torque. The muscle activity of the FPM and PT observed in the present study at the instant of MER, the instant at which the external valgus torque is estimated to be at its maximum during fastball pitching, strengthens the theory that forearm flexor muscles directly counteract the external valgus torque at MER. The *in-vitro* studies in combination with the results of our study may indicate that the UCL might not resist the entire valgus torque by itself, but that the forearm flexor pronator muscles are able to counteract the valgus torque at least partly during baseball pitching as well.

Elbow flexor and extensor muscles, together with the joint articulation, could also indirectly affect the mechanical resistance of the external valgus torque at the elbow during pitching (Van Trigt et al., 2021). *In-vitro* cadaver studies and forward dynamic model studies showed that the biceps and triceps are important in stabilizing the elbow joint (Morrey et al., 1991; Seiber et al., 2009; Rahman et al., 2018). Our results show co-contraction of elbow flexors and extensors at the instant of MER during fastball pitching. To quantify the indirect effect, the CCI index was calculated. It is shown that the CCI index by Rudolph et al. (2000) is best correlated with joint stiffness (Li et al., 2020), and thus in potentially counteracting an external load. The mean CCI at MER in the present study were 0.35 for the biceps-triceps and 0.26 the FPM-ESM. Similar values were found in a study involving the knee joint, which reported the highest CCI values of 0.4 (SD 0.27) during the loading phase in gait (Knarr et al., 2012). The knowledge of *in-vitro* studies and the forward dynamic modeling study in combination with the observed levels of muscle activity of both flexor and extensor muscles at the instant of MER strengthens the theory of elbow muscle co-contraction at the instant of MER having an in-direct effect in counteracting an external valgus torque during fastball pitching in baseball.

The advantage of the CCI applied in the present study is that it considers the magnitude of muscle activity, but the disadvantage is that it calculates the co-contraction only between two muscles instead of all muscles crossing the joint. Using the average muscle activity over the two muscles pairs would result in a biased estimate of the CCI, because muscle sizes and moment arms are not considered. We did not measure the brachialis and brachioradialis, and especially the brachialis might have an important function in counteracting the valgus torque when co-contracting with the extensor muscles, because it is monoarticular, has a small moment arm and a large PCSA.

The literature shows limited information about the anconeus muscle during pitching, and its function is still under debate. It is shown that the anconeus is active in slowly performed elbow extension tasks, but that it has a weak extension function (Miguel-Andres et al., 2017). However, the contribution of the anconeus in explosive movements like baseball pitching is unknown. It could be hypothesized that the anconeus extension contribution becomes more important in explosive movements. Although may be more reasonable, and in line with our results, is that the anconeus might be important in stabilizing the joint via the described indirect effect.

It is not possible to measure muscle force in a non-invasive way. Therefore, the timing of the EMG signals was corrected with 50 ms electromechanical delay (EMD) for each muscle and each participant to represent the muscle activity as an indication of timing of relative muscles force in relation to the timing parameters assessed in the present study (FC, MER, BR). The EMD depends on participants and the type of muscle contraction (Cavanagh and Komi, 1979). In the study of Cavanagh and Komi (1979) it ranges between 35 ms and 77 ms. In addition, they showed that the effect of the muscle type contraction on EMD is subtle, but the EMD between participants showed more variance. Although applying the EMD in a range from 35 to 75 ms changes the magnitude but the muscles still show increased activity in that range (Figures 1, 2), therefore, it will not affect our conclusion that elbow muscles are active and thus able to counteract the external valgus torque.

In this study a considerable difference in magnitude and patterns of EMG between pitchers is found (Figure 4). This could be explained by the fact that this study contains a heterogenous group of pitchers, including different levels of play and age. However, maybe more reasonable in relation with counteracting the external valgus torque is the fact that EMG activity is not directly correlated with muscle force, because EMG does not consider pitcher's muscle properties. For example, pitchers with less muscle activity might have more fast twitch muscles fibers and/or larger PCSA compared to pitchers with more muscle activity. Thus, next to the muscle activity it is important to be aware of the muscle properties in relation to counteracting the external valgus torque.

The (in)direct effect of elbow muscles at MER is important in understanding and preventing pitching related to elbow injuries in baseball. As the external valgus torque at MER is not only resisted by the UCL, but also counteracted by the muscles overlying the elbow joint, it is important to understand the load distribution over these anatomical structures. Therefore, future research should investigate if pitchers with less elbow muscle force at MER are more prone to injury compared to pitchers with more elbow muscle force. Pitching kinematics and kinetics in combination with the use of musculoskeletal models and EMG measurements could help investigate between-pitcher load distribution of the relevant anatomical structures of the elbow in relation to elbow injury risk.

This study shows elbow muscle activity at the critical moment of pitching. Trainers and coaches should be aware of the shielding effect of elbow muscles in preventing pitchers from elbow injuries. They could include strength and coordination exercises in their training program to optimize the elbow muscles function during pitching. In clinical terms, orthopedics should be aware that the elbow muscles can stabilize the joint and might relieve the UCL from maximal stress in overhead sport motions. This knowledge could be used in return-to-sport programs and to prevent athletes from UCL surgeries.

## Conclusion

The flexor and extensor elbow muscles are active at MER, the instant at which the external valgus torque is estimated to be at its maximum, during fastball pitching in baseball. The FPM and PT have a potentially direct effect in helping the UCL to counteract the external valgus torque. Co-contraction of the elbow flexor and extensor muscles indicate a possible in-direct effect in counteracting the external valgus torque. We believe that active muscular contributions counteracting the elbow valgus torque can be presumed to relieve the UCL from maximal stress and is thus of importance in injury risk assessment in fastball pitching in baseball.

## Data Availability Statement

The original contributions presented in the study are included in the article/Supplementary Material. Raw data can be found online at doi: 10.4121/17021966. Further inquiries can be directed to the corresponding author.

## Ethics Statement

This research was conducted in accordance with the Declaration of Helsinki and the local ethics committee of the Technical University Delft approved the research protocol. The patients/participants provided their written informed consent to participate in this study. Written informed consent was obtained from the individual(s) for the publication of any potentially identifiable images or data included in this article.

## Author Contributions

BT, EG, and DV contributed to the conception and design of the work. BT and EG contributed to the data acquisition and analysis. BT, EG, DV, MH, and FH contributed to the interpretation of the data. BT completed the first draft of the manuscript. All authors contributed to the article and approved the submitted version.

## Funding

This work was supported by the NWO Domain Applied and Engineering Sciences (AES) under project number [R/003635]. This NWO-funded project, named Breaking the High Load—Bad Coordination Multiplier in Overhead Sports Injuries part of the Citius Altius Sanius perspective program (Project 7), is a cooperative effort between the Royal Dutch Baseball and Softball Federation (KNBSB), Royal Dutch Tennis Federation (KNLTB), Vrije Universiteit Amsterdam, Delft University of Technology, Milé Fysiotherapy, PitchPerfect, and PLUX.

## Conflict of Interest

The authors declare that the research was conducted in the absence of any commercial or financial relationships that could be construed as a potential conflict of interest.

## Publisher's Note

All claims expressed in this article are solely those of the authors and do not necessarily represent those of their affiliated organizations, or those of the publisher, the editors and the reviewers. Any product that may be evaluated in this article, or claim that may be made by its manufacturer, is not guaranteed or endorsed by the publisher.
