# Supplementary material for: Establishing the Role of Elbow Muscles by Evaluating Muscle Activation and Co-contraction Levels at Maximal External Rotation in Fastball Pitching
Source: Front Sports Act Living. 2021 Nov 30;3:698592. doi: 10.3389/fspor.2021.698592 (PMC8669487; doi:10.3389/fspor.2021.698592)
Supplement: Supplementary file 1 [file Table_1.DOCX]

# Supplementary Material

Table A.1: Maximal voluntary contact (MVC) tests. The gray arrow indicates the applied force direction of the participant. The black arrow indicates the direction of resistance

| **Muscle group** | **Maximal voluntary contraction test** | **Illustration** |
| --- | --- | --- |
| Flexor pronator group (FPM) & pronator teres | Seated or kneeling position in front of a table. With the forearm in approximately 90 °with respect to the upper arm. Participant performs a wrist flexion by pushing the hand palm against the bottom of a ground fixed table. The table functions as static resistance. | 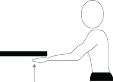 |
| Extensor supinator group (ESM) | Seated or kneeling position in front of the table. With the forearm in approximately 90 °with respect to the upper arm. Participant performs a wrist extension by pushing the back of the hand against the top of the table. The table functions as static resistance. | 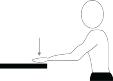 |
| M. biceps brachii | Seated or kneeling position in front of the table. With the forearm in approximately 90 °with respect to the upper arm, and the elbow rests on top of the table. One of the researchers apply a static resistance against the forearm while the participant performs an elbow flexion | 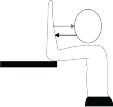 |
| M. triceps brachii & Anconeus | Seated or kneeling position in front of the table. With the forearm in approximately 90 °with respect to the upper arm, and the elbow rests on top  of the table. One of the researchers apply a static resistance against the forearm while the participant performs an elbow extension. | 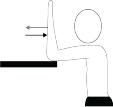 |
