# Supplementary figures and images for: Establishing the Role of Elbow Muscles by Evaluating Muscle Activation and Co-contraction Levels at Maximal External Rotation in Fastball Pitching
Source: Front Sports Act Living. 2021 Nov 30;3:698592. doi: 10.3389/fspor.2021.698592 (PMC8669487; doi:10.3389/fspor.2021.698592)

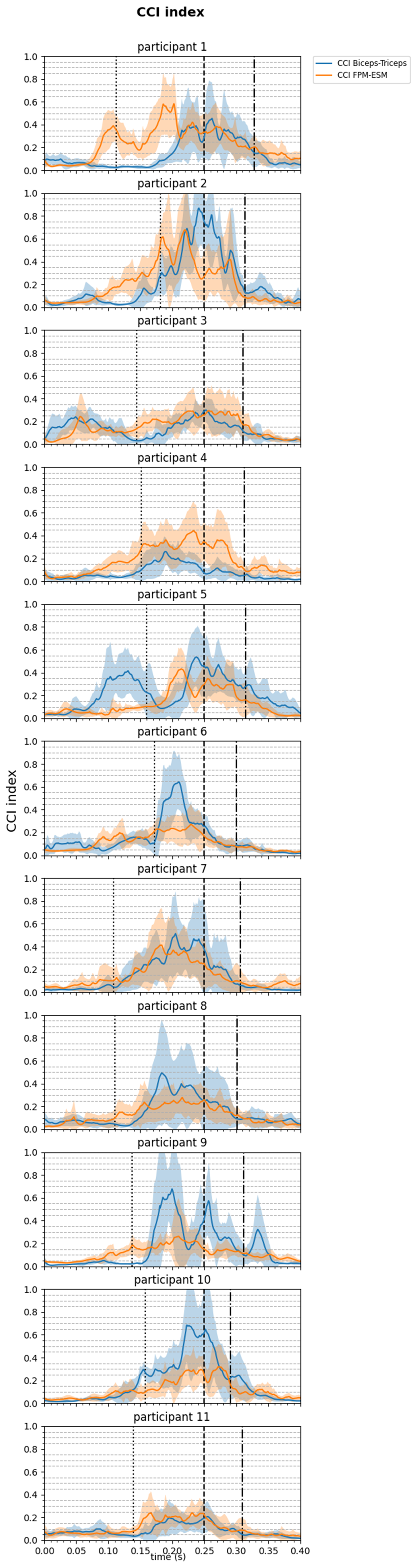

Supplement: Supplementary file 2 [file Image_1.PNG]

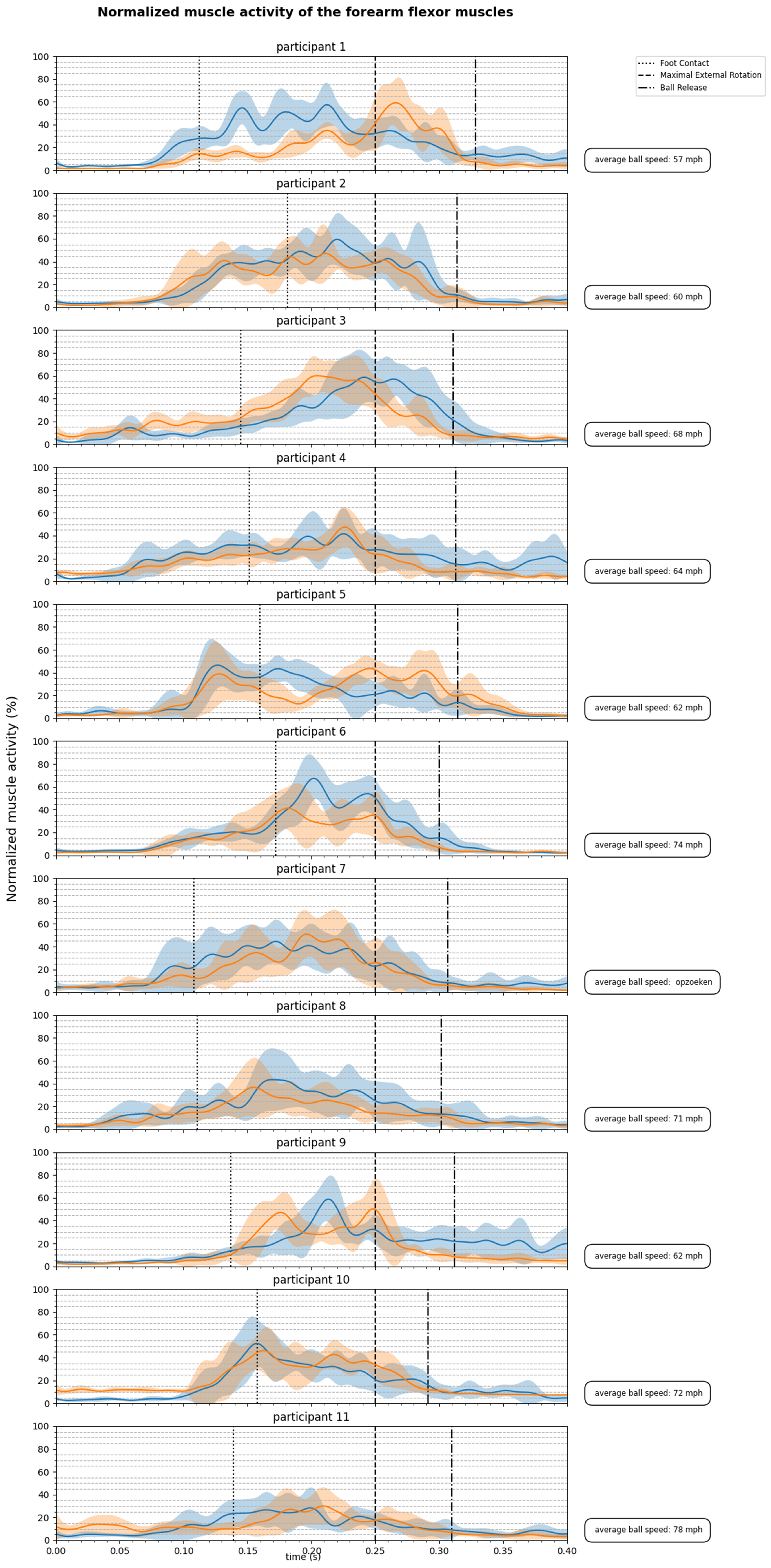

Supplement: Supplementary file 3 [file Image_2.PNG]

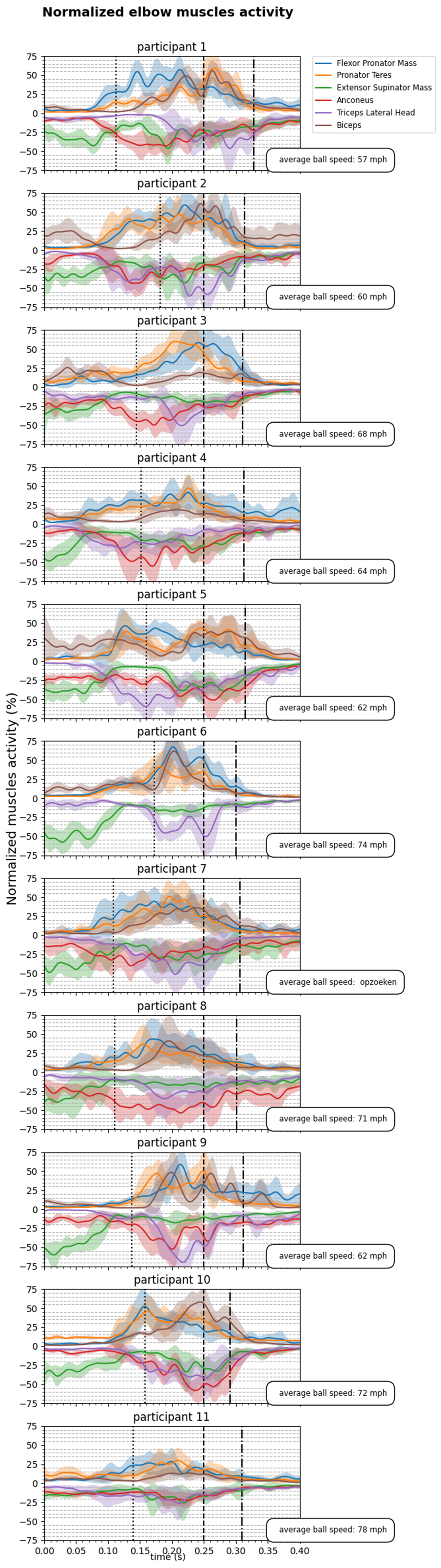

Supplement: Supplementary file 4 [file Image_3.PNG]
